# Development and Decline of Upright Gait Stability

**DOI:** 10.3389/fnagi.2014.00014

**Published:** 2014-02-05

**Authors:** Marco Iosa, Augusto Fusco, Giovanni Morone, Stefano Paolucci

**Affiliations:** ^1^Clinical Laboratory of Experimental Neurorehabilitation, Fondazione Santa Lucia, Istituto di Ricovero e Cura a Carattere Scientifico (IRCCS), Rome, Italy

**Keywords:** locomotion, walking, balance, falls, accelerometry, motor control, aging, neuromuscular diseases

## Abstract

Upright gait is a peculiar characteristic of humans that requires the ability to manage upper body dynamic balance while walking, despite the perturbations that are generated by movements of the lower limbs. Most of the studies on upright gait stability have compared young adults and the elderly to determine the effects of aging. In other studies, the comparison was between healthy subjects and patients to examine specific pathologies. Fewer researches have also investigated the development of upright gait stability in children. This review discusses these studies in order to provide an overview of this relevant aspect of human locomotion. A clear trend from development to decline of upright gait stability has been depicted across the entire lifespan, from toddlers at first steps to elderly. In old individuals, even if healthy, the deterioration of skeletal muscle, combined with sensorial and cognitive performance, reduces the ability to maintain an upright trunk during walking, increasing the instability and the risk of falls. Further, the pathological causes of altered development or of a sudden loss of gait stability, as well as the environmental influence are investigated. The last part of this review is focused on the control of upper body accelerations during walking, a particularly interesting topic for the recent development of low-cost wearable accelerometers.

## Introduction

An experience of great pleasure for a person is surely when his/her child begins to walk independently. Those first few unstable steps are considered a fundamental stage of human development. Arguably, the child’s clear pronunciation of his first word rivals this milestone.

Differently from other animals, human children need more time to develop independent gait (Garwicz et al., 2009). Fourteen months are in mean needed (Bosch et al., 2007) because baby’s rudimentary lower limb flexion–extension movements evolve into sophisticated coordination that permits upright walking (Dominici et al., 2011). Upright walking was not only a fundamental stage in the ontogenesis of a child but also a basic step in phylogenetic human evolution that about 1.5 million years ago evolved to *Homo erectus*. Erectus should not be considered a synonym of bipedal walking but as a stable, upright upper body posture during walking.

The expression “stable gait” may refer to a step-to-step repeatable walking (Ingwell and Marin, 2006), to a gait resilient to external and internal perturbations (Terrier and Dériaz, 2011), or to the ability of maintaining upright balance during walking (Menz et al., 2003). A recent review identified 92 different linear and non-linear quantitative measures of “gait stability” obtained using five different categories of devices and mainly related to spatio-temporal gait parameters, lower limb joint kinematics, and upper body kinematics (Hamacher et al., 2011). About this latter aspect, “upright gait stability” refers to the capacity of humans to minimize oscillations during walking, in a progressive way from the lower to upper levels of the human body, with the head moved on a straight line at quite constant speed (Cappozzo, 1981; Inman et al., 1981). The stabilization of the head during human gait allows steadying the optic flow and increasing the control of equilibrium with more effective processing of vestibular system signals (Berthoz and Pozzo, 1994; Spoor et al., 1994). These and other advantages, such as keeping the hands free and seeing farther, might have spurred the development of upright walking in humans (Friedman, 2006). In general, both exploratory and performative activities can benefit from the upright posture maintained also during gait (Reed, 1996).

The purpose of this review was to investigate the development and the decline of upright gait stability during the lifespan, both in unimpaired and impaired subjects. In the second part of this review, particular attention was deserved to the upright gait stability, i.e., to the upper body dynamic balance evaluated in terms of the capacity of smoothing accelerations during walking.

## Development of Upright Gait Stability

### Physiological development

In typical development, gait mechanism becomes predominantly functional and efficient by the age of 2 years [walking speed (WS) close to 0.8 stature/s (Sutherland et al., 1980) and 60% of energy recovery (Ivanenko et al., 2004)]. However, children may need more time to achieve a stable gait and to manage the emerging instability that accompanies the progressive increase in WS (Iosa et al., 2012f). The beginning of independent walking, averaging at 14 months (Bosch et al., 2007) is characterized by extensive trunk oscillations and a wide base of support, which help the control of instability in new walkers (Ivanenko et al., 2007). At this age, both motor and somatosensory systems need to be developed in order to improve balance and dynamic equilibrium control (Bosch and Rosenbaum, 2010). Unlike the adults, the lateral component of kinetic energy of the center of mass is not negligible, compared to its sagittal components, in toddlers (Ivanenko et al., 2004).

Upper limbs play an important role in this scenario. Healthy adults move their upper and lower limbs in a contralaterally synchronized fashion, to reduce the moment of inertia and maintain upright gait stability (Sparrow and Newell, 1994). At the early stages of gait development, however, healthy toddlers keep their upper limbs high, similar to the spastic limbs observed in children with cerebral palsy. During development, children lower this “high guard” position of the upper limbs, increasing their WS (Kubo and Ulrich, 2006a). The interaction between arm posture and upper trunk position creates a variety of changes in forces and torque between individuals that can be explained by the need for healthy toddlers to explore their dynamics and gait stability in the early stages of gait development (Pozzo et al., 1990).

From age 7, upper body gait stability is related to the adoption of head stabilization (Pozzo et al., 1990), which appears to be more difficult in the latero-lateral (LL) compared to the antero-posterior (AP) direction (Assaiante and Amblard, 1993; Mazzà et al., 2010). At age 9, children show upper body stability, similar to that of adults (Mazzà et al., 2010). Unfortunately, few data are available about individuals between 10 and 20 years of age, preventing a detailed examination on what occurs in teenagers: in this age, cultural habits and psychological features may be also have an impact on functional development and anthropometric growth.

### Altered development

In children with impaired development, such as those with cerebral palsy, the ability to walk at a proper speed is limited in the first phase of independent walking, even if they are not severely affected (Iosa et al., 2012f). Later, they develop a faster speed, implying high trunk instability (Iosa et al., 2012f). Higher vertical oscillations in these children are likely related to their equinus foot or crouch gait, two typical features of cerebral palsy (Perry, 1992; Gage, 1993). Along LL direction, an increased distance has been found between the body center of mass and the center of pressure under the foot of standing leg in children with diplegia due to cerebral palsy during single support phase of gait (Hsue et al., 2009a,b). This could be due to three possibly concomitant reasons: (1) hip muscle weakness (see Gait Instability and Risk of Fall for details); (2) facilitating contralateral foot lift; (3) facilitating the control of forward progression by transferring part of forward momentum to lateral momentum.

The high trunk accelerations and gait asymmetry that are observed in children with cerebral palsy are likely to be related to certain compensatory mechanisms that facilitate walking at a speed that is similar to that of unimpaired children (Meyns et al., 2012). Greater arm swing on the unaffected side is often required to compensate for the reduced movement on the affected side and to counteract the substantial increases in angular momentum that are generated by functional asymmetry in the legs (Bruijn et al., 2011). Thus, it is conceivable that this compensatory strategy destabilizes gait patterns (Bruijn et al., 2011), reducing upright gait stability (Iosa et al., 2012f).

In children with Down syndrome, the development of independent walking usually occurs approximately 1 year later than in children who undergo typical development (Henderson, 1986). At the early stages of walking, children with Down syndrome have similar gait spatio-temporal patterns as typically developed toddlers but appear to be less able to manage the coupling of AP–LL oscillations at the level of the center of mass, i.e., at the lower trunk level (Kubo and Ulrich, 2006b).

The altered development of upright gait stability is an interesting topic, and further studies are needed to examine the development of gait stability in toddlers with and without developmental disability. The possibility of easily assessing gait related features by wearable devices (rather than with more complex stereophotogrammetric systems) might be helpful for early clinical analysis and, in general, for a greater understanding of this processes (Masci et al., 2013).

## Decline of Upright Gait Stability

### Physiological decline with aging

Many studies reported a decline of gait stability in the elderly. Experimental evidence shows that this reduction in the ability to stabilize the upper body during walking is due to the loss of skeletal muscle strength (Doherty, 2003) and a reduction in the ability to detect and process proprioceptive and sensorial information (Scaglioni et al., 2003).

From a motor point of view, after the age of 50, approximately 1–2% of muscle mass is lost per year. This muscle mass and strength reduction, called sarcopenia, is also accompanied by intramuscular fat accumulation, muscle atrophy (especially the type IIa fibers), decreased satellite cell proliferation and differentiation capacity, and reduction in motor unit number (Muscaritoli et al., 2013). The loss in body mass density, which is related to muscle weakness, is greater in women compared to men aged 60 years and older (Daly et al., 2013). From a skeletal point of view, the age-related degradation of joints, including articular cartilage and bone epiphysis, especially at knee level, can cause gait impairments, especially when it suddenly degenerates in pathological osteoarthritis (van Baar et al., 1998).

A recent study had also suggested that vitamin D may be involved in gait stability. Vitamin D metabolism is in fact involved in muscular contraction speed and one of the consequence of its concentration reduction, typically occurring in the elderly, was found to be an increased stride-to-stride duration variability that is related to a reduction of gait stability and hence to an increased risk of fall (Beauchet et al., 2011).

Aging implies not only a loss in musculoskeletal functioning, but also a decline in vision, reaction time, peripheral and vestibular sensations: all of which can reduce upper body stability during walking (Menz et al., 2003). The vestibular system becomes impaired with age, altering the vestibulospinal reflex function (Allum et al., 1997). With regard to the chief function of the vestibular system in upright gait stability, it is noteworthy that the inner ear of *Homo erectus* was more developed than in earlier hominids, who possessed ape-like inner ears (Spoor et al., 1994).

Considering these reduction in sensorimotor ability, older persons have a more conservative gait pattern, characterized by reduced velocity that is a result of a shorter step length and accompanied by increased variability in step timing (Menz et al., 2003). These differences are particularly pronounced when they walk on irregular surfaces (Menz et al., 2003; Marigold and Patla, 2008). The conservative basic gait patterns, evident in older people, might be a compensatory strategy to ensure adequate stabilization of the upper body (Menz et al., 2003). It has been proposed that these gait patterns in older people are attributed in part to a decline in leg strength and a reluctance to walk quickly, so that their upper body accelerations do not increase (Menz et al., 2003). Notably, the elderly experience an abrupt increase in instability in the LL direction. These results are also supported by the excessive lateral momentum found in balance-impaired during gait (Kaya et al., 1998) and by the increased center of mass – center of pressure inclination angle measured in lateral direction (frontal plane) (Lee and Chou, 2006) that were both found as sensitive measures of loss of equilibrium in elderly during gait. Lateral trunk bending has been described as a typical compensation mechanism for hip abductor weakness: lateral trunk movements toward the stance limb align the body’s center of mass over the hip joint center, supporting the body in the single stance when the hip abductor muscles are weak (Perry, 1992). This trunk bending strategy, helpful for compensation hip muscle weakness, is typical not only of old age, but also of many pathologies (as described below), including children with cerebral palsy with lateral excessive trunk bending, as reported above.

Not only motor and sensorial impairment have been shown to affect upright gait stability in the elderly. Indeed, physiological reduction of cognitive functions might also have a significant role, as suggested by studies that reported decreased gait stability in subjects with cognitive impairment (see Pathological Loss of Gait Stability) (Lamoth et al., 2011; Ijmker and Lamoth, 2012). This topic appears rather controversial, as the study entitled “walking without thinking” (Ruchinskas et al., 2000) questions the role of cognitive function on gait control, observing that, while it could affect the overall geriatric rehabilitation course, it may not predict walking or stair climbing ability. It must be remembered, however that cognitive status and thinking are two different things. Another study, in fact, observed that in elderly persons without cognitive impairment, gait patterns were altered when subjects were asked to walk and perform a cognitive naming task simultaneously (Lamoth et al., 2011). In response to dual tasking, subjects decreased their WS by increasing stride time. Despite the lower speed, trunk acceleration patterns were more irregular and variable, and local stability declined in the dual task condition. These results highlight the importance to take into account the control of functional resource allocation, with a reduction in upright gait stability when subjects are asked to walk and perform a cognitive task simultaneously, as occurs often in everyday life.

Finally, upright gait stability has been found adversely associated to subclinical cerebrovascular lesions. Silent infarcts (detectable in 7.2–28% of the general population, i.e., in the absence of a history of clinical stroke), cerebral microbleedings (occurring in 4.7–23.5% of the general population), and cerebral white matter microlesions (present in 90% of people aged >60 years) affect gait ability and stability in elderly (Choi et al., 2012).

### Gait instability and risk of fall

All the difficulties described above in controlling dynamic balance during walking in the elderly have also been associated with the risk of falls (Marigold and Patla, 2008; Senden et al., 2012). There are currently over 400 known risk factors for falls, broadly classified into environmental, task-related, and personal factors (Masud and Morris, 2001). Age is one of the most important risk factor, and many other personal factors may be related to age: among others, muscular strength, reaction time, visual ability, use of drugs and medications, living alone, sedentary behavior, psychological status, and impaired cognition and foot problems (Hamacher et al., 2011). As above reported, most of them are also associated to higher upper body accelerations and higher step-by-step gait variability.

Environmental, task-related, and personal factors may also increase the risk of fall. For example, this risk increases when older individuals can not reduce their WS by controlling their trunk acceleration. This event may occur when they want to cross a street and must time their gait speed with the traffic lights, which usually requires a mean speed of at least 0.9 m/s (Hesse et al., 2009), higher than what many older subjects can attain (see Table 1; Mazzà et al., 2008; Lamoth et al., 2011; Senden et al., 2012). For example, in UK, such as in other Western countries, 1.2 m/s is the value of theoretical WS utilized to set the crossing timing of traffic lights (Asher et al., 2012). However, the comfortable speed of many elderly is lower than these values (Asher et al., 2012). A speed lower than 0.8 m/s has been defined as a pathological gait velocity and resulted associated with repeated falls, even when observed in healthy elderly (without any specific pathology) (Montero-Odasso et al., 2004). But a low steady speed is not the only factor affecting the safety in road crossing. The presence of obstacles (Hahn and Chou, 2004), the visual attention and the time-to-arrival estimation (Dommes and Cavallo, 2011), the fear of falling (Avineri et al., 2012), the time spent in selecting the proper motor strategy (Sueur et al., 2013), as well as the management of accelerations (for example in the start of walking, or for further increasing speed if traffic green light is going to expire or if a car is arriving) or deceleration for terminating road crossing (Bollard and Fleming, 2013) are all factors that can reduce gait stability and increase the risk of fall or of an accident.

**Table 1:** **Healthy subject trunk accelerations during walking ordered by age**.

Authors (year)	Age (years)	WS (m/s)	Gender	Trunk level	*n*	RMS_AP_ (m/s^2^)	RMS_LL_ (m/s^2^)	RMS_CC_ (m/s^2^)
Iosa et al. (2012f)	4 ± 1	0.94 ± 0.22	M and F	L2–L3	8	2.23 ± 0.60	2.14 ± 0.47	2.95 ± 0.81
	7 ± 2	1.09 ± 0.13	M and F	L2–L3	9	1.72 ± 0.40	1.54 ± 0.48	2.44 ± 0.86
Mazzà et al. (2010)	9 ± 1	1.34 ± 0.14	M	Pelvis	15	1.93 ± 0.31	1.76 ± 0.23	2.90 ± 0.67
	9 ± 1	1.32 ± 0.16	F	Pelvis	15	1.97 ± 0.31	1.79 ± 0.31	2.93 ± 0.85
Mazzà et al. (2009)	23 ± 2	1.33 ± 0.13	M	Pelvis	20	1.91 ± 0.40	1.57 ± 0.36	2.70 ± 0.72
	23 ± 3	1.34 ± 0.09	F	Pelvis	20	2.06 ± 0.40	1.90 ± 0.22	2.88 ± 0.13
Moe-Nilssen (1998)	23 ± 2	1.2^a^	M and F	L3	19	1.57 ± 0.18	1.33 ± 0.17	2.06 ± 0.23
Kavanagh (2009)	23 ± 3	1.32 ± 0.18	M and F	L3	13	1.63 ± 0.17	1.23 ± 0.16	1.91 ± 0.23
Iosa et al. (2012b)	28 ± 5	1.12 ± 0.11	M and F	L2–L3	28	1.72 ± 0.28	1.51 ± 0.36	2.76 ± 0.56
Iosa et al. (2010)	31 ± 9	1.25 ± 0.08	M and F	Pelvis	13	1.53 ± 0.25	0.56 ± 0.07	1.79 ± 0.35
Henriksen et al. (2004)	35	1.35^a^	M and F	L3	20	1.75 ± 0.20	1.35 ± 0.25	2.47 ± 0.24
Iosa et al. (2012d)	63 ± 10	1.17 ± 0.18	M and F	L2–L3	10	1.49 ± 0.30	1.02 ± 0.22	2.21 ± 0.70
Iosa et al. (2012e)	29 ± 5	1.13 ± 0.11	M and F	L2–L3	15	1.75 ± 0.35	1.57 ± 0.39	2.87 ± 0.65
	65 ± 9	1.02 ± 0.16	M and F		15	1.37 ± 0.26	0.96 ± 0.22	1.94 ± 0.54
Mazzà et al. (2008)	24 ± 4	1.30 ± 0.28	F	Pelvis	16	1.41 ± 0.23	0.48 ± 0.18	1.66 ± 0.92
	72 ± 4	0.97 ± 0.18	F	Pelvis	20	1.06 ± 0.24	0.48 ± 0.14	0.99 ± 0.32
Kavanagh et al. (2004)	23 ± 4	1.28 ± 0.15	M and F	L2–L3	8	1.47 ± 0.98	1.10 ± 0.78	1.77 ± 1.17
	74 ± 3	1.23 ± 0.15		L2–L3	8	1.37 ± 0.98	1.10 ± 0.69	1.77 ± 1.10
Marigold and Patla (2008)	26 ± 5	1.0^a^	M and F	Iliac crests	10	0.95 ± 0.10	0.57 ± 0.09	1.65 ± 0.21
	74 ± 7				10	1.05 ± 0.16	0.74 ± 0.13	1.56 ± 0.17
Ijmker and Lamoth (2012)	64 ± 3	1.19 ± 0.08	M and F	L3	12	1.40 ± 0.16	1.28 ± 0.19	Not eval.
	77 ± 4	1.14 ± 0.11			14	0.84 ± 0.32	0.95 ± 0.31	
Lamoth et al. (2011)	79 ± 5	0.95 ± 0.21	M and F	L3	13	1.04 ± 0.23	0.96 ± 0.18	Not eval.
Menz et al. (2003)	29 ± 4	1.33 ± 0.19	M and F	Pelvis	30	1.86 ± 0.39	1.86 ± 0.39	2.55 ± 0.69
	79 ± 3	1.17 ± 0.16		Pelvis	30	1.67 ± 0.29	1.57 ± 0.49	1.96 ± 0.49
Senden et al. (2012)	74 ± 5	1.23 ± 0.22	M and F	Sacrum	50	Not eval.	Not eval.	2.45 ± 0.69
	79 ± 6	0.86 ± 0.26			50			1.57 ± 0.69

*The columns report authors and year of publication, the reference number for each study, the mean age (±SD) of groups of subjects, walking speed (^a^ in some studies a reference value of WS has been used to identify trunk acceleration from interpolant curves), gender, analyzed level of trunk (in many of these studies, other parts of the body are analyzed), number of subjects enrolled (*n*), and mean acceleration RMS along the antero-posterior (AP), latero-lateral (LL), and cranio-caudal (CC) axis. In some of the original studies, accelerations were reported in *g*, in which case they were transformed to m/s^2^, or reported in terms of mean and standard error of the mean, in which case they were transformed to mean ± SD. For study Henderson (1986), the data are related to the first 6 min of walking. Finally, not all studies evaluated accelerations along all three body axes*.

Regarding acceleration, upper body cranio-caudal (CC) accelerations were found significantly increased in elderly with balance impairment compared to elderly without balance impairment (Senden et al., 2012). Furthermore, it has been reported that the harmonicity of trunk movements (assessed by analysis of trunk accelerations in the domain of frequency) is a reliable predictor of the risk of falls in elderly, independently of their physical performance, in 1 year after the assessment (Doi et al., 2013) (for details about these last two studies, see Assessment of Dynamic Equilibrium by Trunk Accelerations).

A recent review analyzed the available kinematic measures for assessing gait stability, taking into account old versus young subjects’ comparisons and fallers versus no-fallers comparisons (Hamacher et al., 2011). The authors found that, regarding the 92 different outcome measures identified in literature, linear measures of gait stability showed a higher sensitivity concerning age-related differences than non-linear measures. In detail, variability of step width and stride velocity were capable of distinguishing between old and young subjects, whereas variability of stride, stance, and swing between fallers and non-fallers. Step width variability had higher discriminative power in the comparison between elderly and young subjects than in the faller versus non-faller comparisons, suggesting that although it may increase with age, it is not necessarily a dominant factor in fall risk. This suggestion has also been supported by the fact that differences between fit and frail older adults were better highlighted by interstride trunk acceleration variability, than by step width variability (Moe-Nilssen and Helbostad, 2005). Another study reported increased trunk acceleration and trunk roll variability along LL direction in elderly walking, despite having similar step width of the control group formed by younger subjects (Marigold and Patla, 2008).

According to all these studies, the increased LL trunk acceleration observed in advanced age seems to be a particularly important risk of fall given its association with gait instability. Moreover, hip fractures occur most frequently in association with lateral falls.

### Pathological loss of gait stability

The loss of stability can be abrupt when a pathological event occurs, wherein more severe pathologies affect a greater loss of upright gait stability.

Upright gait stability has been studied extensively in subjects after stroke. In these patients, upper body accelerations are significantly lower than in age-matched healthy controls in all axes due to the reducing WS. However, acceleration root mean square (RMS) values that have been normalized by speed are higher than in healthy subjects along all body axes in subjects with chronic (Mizuike et al., 2009) and subacute stroke (Iosa et al., 2012e). As shown in Table 1, the mean WS of young healthy adults was about 1.3 m/s. Although WS in stroke patients (reported in Table 2) declines by approximately 50% versus young healthy adults, their CC acceleration (in terms of its RMS), their AP and LL accelerations do not. Yet, in patients with stroke, trunk control is a prognostic factor for gait recovery (Masiero et al., 2007; Di Monaco et al., 2010): their trunk asymmetries are more marked than their asymmetries with regard to step length and single support timing (Hodt-Billington et al., 2008).

**Table 2:** **Gait stability data of subjects with a pathology or healthy subjects walking on uneven floor or without vision support, with respect to reference values obtained as the average of means (±SDMs) reported in studies on young healthy adults**.

Condition	Reference	Age (years)	WS (m/s)	Trunk level	*n*	RMS_AP_ (m/s^2^)	RMS_LL_ (m/s^2^)	RMS_CC_ (m/s^2^)
Cerebral palsy	Iosa et al. (2012f)	4 ± 1	0.76 ± 0.19	L2–L3	11	2.29 ± 0.78	2.26 ± 0.93	3.04 ± 1.43
		7 ± 2	1.15 ± 0.09	L2–L3	6	2.86 ± 0.91	2.50 ± 0.68	4.29 ± 1.31
Stroke	Iosa et al. (2012e)	61 ± 15	0.60 ± 0.29	L2–L3	15	0.98 ± 0.33	0.80 ± 0.24	1.16 ± 0.50
	Iosa et al. (2012d)	64 ± 13	0.55 ± 0.31	L2–L3	20	0.91 ± 0.37	0.87 ± 0.41	1.13 ± 0.61
Dystrophy	Iosa et al. (2010)	39 ± 11	1.04 ± 0.15	Pelvis	13	1.31 ± 0.36	0.59 ± 0.16	1.30 ± 0.40
Dementia	Lamoth et al. (2011)	83 ± 4	0.88 ± 0.27	L3	13	1.03 ± 0.26	0.82 ± 0.18	Not eval.
	Ijmker and Lamoth (2012)	82 ± 6	0.67 ± 0.21	L3	15	0.56 ± 0.22	0.59 ± 0.21	Not eval.
Uneven floor	Moe-Nilssen (1998)	23 ± 2	1.2^a^	L3	19	1.72 ± 0.22	1.57 ± 0.18	2.28 ± 0.23
No vision	Iosa et al. (2012b)	28 ± 5	0.83 ± 0.18	L2–L3	28	1.32 ± 0.36	1.23 ± 0.36	1.87 ± 0.68
Reference values	Mazzà et al. (2009), Iosa et al. (2012e), Menz et al. (2003), Kavanagh et al. (2004), Moe-Nilssen (1998), Henriksen et al. (2004), Marigold and Patla (2008)	26 ± 4	1.26 ± 0.10	Lower trunk	183	1.67 ± 0.30	1.40 ± 0.39	2.36 ± 0.47

*^a^A reference value of WS from interpolant curve*.

Overexertion and fatigue can compromise the control of gait stability following stroke. Two alternative strategies have been highlighted in these patients for prolonged (6-min) walking. Some subjects maintain their speed during long-lasting walking, despite a slight but progressive reduction in upper body stability. Other subjects apply a compensatory strategy, based on a reduction in WS for maintaining low upper body accelerations (Iosa et al., 2012d).

Patients with Parkinson’s disease show increased stride time variability, reduced trunk rotation, reduced arm swing as signs of gait impairments as well as increased time spent in the double support phase that is probably due to the need of improving dynamic equilibrium during gait (Horak and Mancini, 2013). l-DOPA reduces stride time variability, decreases double support time, and increases gait speed, all consistent with improvements in gait dynamic balance (Rebula et al., 2013). Interesting studies showed a reduced coordination between the rhythmic processes of the two legs in these patients (Plotnik et al., 2007). In particular, reduced inter-lower limb coordination and higher stride time variability can expose these subjects to a higher risk of falls. Furthermore, during the execution of a cognitive task while walking, gait symmetry was reduced in these patients (Plotnik et al., 2011).

Subjects who are affected by muscular impairment, such as dystrophy, have wider and less symmetrical upper body oscillations than healthy controls in the AP and LL directions. Further, the ability of these subjects to attenuate accelerations from the lower body to the head is weakened. It has been suggested that these features are related not only to upper body muscle impairments but also indirectly to the above described strategy that compensates for hip muscle weakness (Iosa et al., 2010).

As above mentioned, increased LL trunk bending, as described in the elderly, is a typical compensatory mechanism for hip abductor weakness also in other pathologies, such as dystrophy (Iosa et al., 2010), myelomeningocele (Gutierrez et al., 2005), and spinal muscular atrophy (Armand et al., 2005).

Upright gait stability in patients with vestibular deficits is a notable area of study. However, most researches on such patients have investigated their postural standing stability, instead of gait dynamic stability. Nevertheless, one of the few studies on upper body stability during walking in persons with vestibular hypofunction reported lower regularity of upper body movements compared with healthy subjects, consistent with the function of the vestibular system in controlling the dynamic stability of walking (Sylos-Labini et al., 2012). Also, in patients with small vestibular schwannomas and an apparently normal gait who are asked to walk without vision support, gait variability increases, reducing their gait steadiness (Yin et al., 2011). After unilateral vestibular neurotomy, used as curative surgery in patients with Menière syndrome, robust walking deviations develop toward the damaged side on walking with the eyes closed (Borel et al., 2004).

Another sensorial impairment that affects gait stability is altered vision (Perry et al., 2001). However, few studies have examined upright gait stability in a population with visual impairment. Yet, significant differences exist between subjects with and without visual impairments and between conditions of full vision and no vision. Further, these differences reflect a more cautious walking strategy and adaptive changes under challenging conditions. In particular, subjects with visual impairments undergo adaptations that are related to a shorter stride length and prolonged plantar foot contact. These patterns might reflect a strategy to overcome the problems that arise from sensory deprivation, compensated by the use of the feet to probe the ground for haptic exploration, to maintain adequate upper body stability (Hallemans et al., 2010).

In less disabling pathologies, such as low back pain, subjects exhibit altered coordination of the pelvis and trunk, resulting in a less harmonic and less stable gait (Lamoth et al., 2002).

Finally, reductions in cognitive functions, such as those that are related to dementia (Allali et al., 2008; Lamoth et al., 2011; Ijmker and Lamoth, 2012) and Alzheimer disease (Sheridan et al., 2003), contribute to changes in the variability and stability of the gait pattern when the task becomes more challenging, increasing the risk of falls.

## Other Factors Influencing Upright Gait Stability

Upright gait stability also depends on the capacity of subjects to exploit information on the orientation of the swaying body during walking with respect to the environment. This information is provided primarily by the vestibular system, lower-extremity mechanoreceptors, and vision (Lee, 1980; Perry et al., 2001), but also environmental features can affect upright gait stability (Iosa et al., 2012b) and hence the risk of fall (Hamacher et al., 2011). For example, under impaired physical conditions, subjects usually adopt a strategy to reduce velocity in challenging environments, decreasing upper body accelerations.

A typical situation is when one walks on uneven ground, implying greater upper body acceleration, even in young healthy adults (Moe-Nilssen, 1998). Also, a sudden reduction in environmental light implies an increase in trunk accelerations and inter-step trunk acceleration variability (Moe-Nilssen et al., 2006). Conversely, optic flow can alter locomotion parameters (Pailhous et al., 1990) and mediates the maintenance of a steady gait (Pailhous and Bonnard, 1992).

When vision is blocked completely, the stability, in terms of normalized accelerations, decrease drastically and the harmony of movements in the AP direction falls significantly (Iosa et al., 2012b). These findings demonstrated the fundamental role of vision in upright gait stability, confirming that vestibular, proprioceptive, acoustic, and tactile information can not fully compensate for the loss of visual information to produce a normal gait pattern (Hallemans et al., 2009; Iosa et al., 2012b). There is also a slight difference between walking outdoors and indoors, with higher AP accelerations outdoors, a difference that increases in the absence of visual feedback (Iosa et al., 2012b). It has been suggested that when visual information is lacking, the memorized environment acts as a selective tuner between various walking strategies that is based more on sensory feedback versus on an internal representation of the own body and the external world (Iosa et al., 2012c).

Upper body stability during walking can be also affected by cultural habits, as already suggested (Mazzà et al., 2009), but this relationship has not been investigated in detail. Walking with books over the head was a common exercise for young aristocratic ladies. Certain gender differences in upright gait stability have recently been studied: females have better control strategy, allowing them to reach head accelerations that are equivalent to those of males, despite having higher LL pelvis accelerations (Mazzà et al., 2009). However, this difference was not found significant at the level of L2–L3 (Iosa et al., 2012b), of shoulders and head (Mazzà et al., 2008). Furthermore, the most of the studies did not separate data between males and females. Nevertheless, gender-related features of upright gait stability are reported and the absence of visual feedback exacerbates these gender-based differences in LL movements (Iosa et al., 2012b). The development of upright gait could have had sexual implications: it has even been suggested that permanent sexuality that replaced female estrus, was an effect of the phylogenetic development of upright gait (Graslund, 1995).

Finally, it is noteworthy that in some areas of the world that lack a transportation infrastructure, people routinely carry extraordinary loads supported by their heads, for example the sherpa of the Himalayas and the women of East Africa. It needs a very stable head during walking and it is a result of a greater conservation of mechanical energy resulting from an improved gait control (Heglund et al., 1995).

Thus, it is conceivable that differences in upright stable gait could be related not only to differences in motor abilities, but also to the dictates of social and cultural prescriptions specific for each phase of life and probably also to geographical differences. These hypotheses, however, are unconfirmed and should be further investigated.

## The Control of Upper Body Accelerations during Gait

### Assessment of dynamic equilibrium by trunk accelerations

In the early 1990s, gait analysis was focused on hip, knee, and ankle angular kinematics, and the mainstream idea was that the upper body was a static passenger unit of a locomotor apparatus that was located primarily at the lower limb level (Perry, 1992). This idea was then challenged by empirical evidence. In fact, many following studies confirmed as the trunk plays a fundamental dynamic role during walking, attenuating acceleration, ensuring the upright posture, and stabilizing the optic flow and vestibular signals (Winter, 1995; Menz et al., 2003; Kavanagh et al., 2004). This stability is achieved through a combination of passive mechanical damping and active feed-forward control of paraspinal muscles (Prince et al., 1994).

According to these studies, interest in upper body movements during walking has even increased in the past decade. A large and growing body of literature has investigated movements of the pelvis, center of mass, trunk, shoulder, and head during walking, primarily for two reasons: the function of upper body during gait and the development of wearable wireless accelerometers for quantifying gait stability. Accelerometers are usually small, lightweight, wireless, and wearable, allowing subjects to walk relatively unrestricted and without the limitations of the test environment of a laboratory (Kavanagh and Menz, 2008; Iosa et al., 2012a).

The second part of this review is mainly focused on the stability of upper body during gait in terms of control of upper body accelerations during gait.

In a 2008 review, Kavanagh and Menz (2008) analyzed 33 reports on the use of accelerometry for gait analysis, noting that the most common location for these devices was the lower trunk (12 of 33 studies, 36%), followed by head (21%) and lower limbs (21%). The authors concluded that the available literature indicates accelerometry as an accurate and reliable measure of basic spatio-temporal gait parameters and segmental accelerations of the body when walking, and a suitable measure for highlighting age-related differences in dynamic balance control during gait in people with movement disorders.

**Figure 1:**
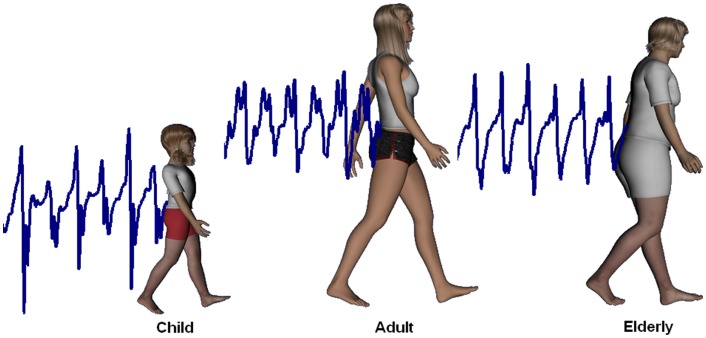
**Upper body accelerations**. Upper body accelerations along the antero-posterior direction in a child (data obtained for a 2-year-old female), an adult (35 years, female), and an elderly subject (69 years, female).

Examples of accelerometric signals that are obtained during walking are reported in Figure 1 for a child, an adult, and an elderly subject along the AP direction. It is possible to observe how upper body AP acceleration was higher and less repeatable in the child and elderly, whereas it was lower and less variable in the young adult.

The root mean square of an acceleration signal is the most commonly used parameter in assessing upper body stability using accelerometric-based gait analysis (Menz et al., 2003; Kavanagh et al., 2004; Marigold and Patla, 2008; Mazzà et al., 2008; Mizuike et al., 2009; Lamoth et al., 2011; Iosa et al., 2012e). It simply corresponds to the standard deviation of acceleration signals at which it has been removed its mean, indicating the dispersion of acceleration. Triaxial accelerometers allow one to measure accelerations along the three body axes: AP, LL, and CC, so the RMS can be evaluated along each one of the three body axes (RMS_AP_, RMS_LL_, RMS_CC_, respectively).

Since the 90s, it has been suggested that RMS of accelerations could be a valid parameter for assessing balance during walking, differentiating between subjects with and without balance impairment or between walking on solid and soft terrains (Moe-Nilssen, 1998). These results were then confirmed by two studies of Menz et al. In the first one, the authors showed the age-related differences in walking stability measured using accelerometers fixed to head and trunk of subjects (Menz et al., 2003). In the second one, they showed as the RMS of pelvis accelerations increases when subjects were asked to walk on irregular surfaces (Menz et al., 2003). Subsequently, many other studies showed as acceleration RMS was a suitable parameter for discriminating between patients with unstable gait and healthy subjects (Mizuike et al., 2009; Lamoth et al., 2011; Iosa et al., 2012e). High reliability of three dimensional accelerometric approach at head, neck, trunk, and shank level was found in test–retest conditions (a mean coefficient of multiple determination across all conditions of 0.87) (Kavanagh et al., 2006).

Senden et al., enrolled 100 elderly subjects that were divided into two groups: with and without unspecific balance disorders clinically assessed by Tinetti scale. Many gait parameters were found correlated with the score of Tinetti scale, but only WS, step length, and acceleration RMS showed moderate to strong correlations and high discriminative power to classify elderly according to their clinical balance assessment. As reported in Section “Gait Instability and Risk of Fall,” another study conducted using an accelerometric-based gait analysis found that the harmonic ratio of trunk acceleration (a parameter extracted by frequency analysis on accelerometric signals) may predict the risk of falls in elderly, independently of their physical performance, in 1 year after the accelerometric assessment (Doi et al., 2013). The harmonic ratio of trunk acceleration was taken into account also in the study of Senden et al. It showed moderate association with the Tinetti score, but did not enter into the final regression model for identifying subjects with unstable gait (Senden et al., 2012). All these studies showed as the analysis of trunk accelerations can be informative about the excessive and disperse trunk movements strictly inter-connected with upright gait instabilities. The relationship between gait harmony and risk of falls needs further studies, however it has recently been suggested that the involuntary control of locomotion can be favored by the intrinsic gait temporal harmony, implying that its lost can increase the difficulties in controlling gait (Iosa et al., 2013).

Several studies have advocated normalizing upper body acceleration as a function of gait speed (Marigold and Patla, 2008; Kavanagh, 2009; Iosa et al., 2012e). In fact, increasing or decreasing gait speed effects a corresponding quadratic rise or decline in acceleration amplitude, thus, RMS values need to be normalized between subjects and populations at different walk speeds to assess only the upper body dynamic instabilities that are imputable to some balance impairment suitably (Mizuike et al., 2009; Iosa et al., 2012e). To take into account the relationship between acceleration RMS and WS, many different methods have been suggested (Moe-Nilssen and Helbostad, 2004; Marigold and Patla, 2008; Mizuike et al., 2009; Iosa et al., 2012e,f). Clinicians should hence keep clearly in mind that an increase in upper body accelerations could be attributed to an unsteady speed due to gait instabilities or a rise in WS, in absence of a suitable normalization of accelerometric values (Iosa et al., 2012e).

In this last part of the review, we summarize the results of the studies that compared upright gait stability between elderly and young adults or between subjects with a specific pathology or impairment and age-matched healthy controls, providing an overview of the development and the decline of upright gait stability in terms of upper body acceleration control.

### Development and decline of upper body acceleration control during gait

Table 1 reports the results of 15 studies regarding the measurements of upper body accelerations in 552 healthy subjects during walking. Only the data related to healthy subjects enrolled in these studies are reported in the table, and they were ordered by age. Most of these studies used accelerometers to assess upper body gait stability; two studies used stereophotogrammetry (Mazzà et al., 2008; Iosa et al., 2010) and one used an optoelectronic system (Marigold and Patla, 2008). The stereophotogrammetric analysis studies generated lower values of acceleration RMS along the LL direction compared with other studies, probably because analyzing a virtual point inside of the body, rendering them less sensitive to rotations around the CC axis.

**Figure 2:**
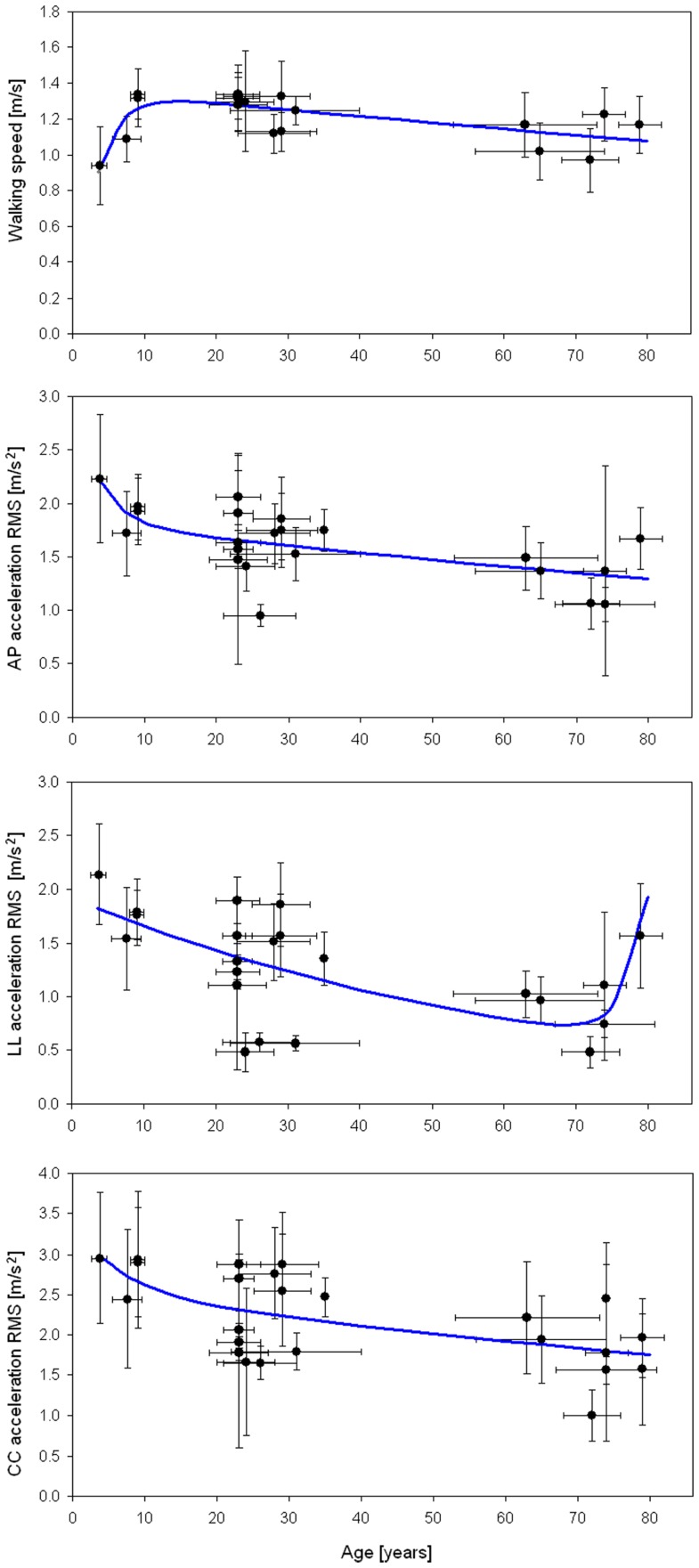
**Walking speed and acceleration RMSs**. Mean (±SD) of walking speed, AP, LL, and CC acceleration RMSs with respect to mean (±SD) age for the studies in Table 1. Regression lines (in blue) were obtained with a bi-exponential fit.

Many of these studies compared upper body accelerations between healthy subjects (reported in Table 1) and patients. Figure 2 graphs the results of the studies in Table 1. WS and RMSs were found significantly correlated with age (Pearson correlation: *p* = 0.031 for WS, *p* < 0.001 for RMS_AP_, *p* = 0.013 for RMS_LL_, *p* = 0.003 for RMS_CC_). However, bi-exponential curves better fitted the data than linear regression fits (mean coefficient of determination 0.43 versus 0.30). The equation of the bi-exponential fit, applied using the least mean square method, was: $y=a\cdot e^{-\frac{x}{\tau_{1}}}+b\cdot e^{-\frac{x}{\tau_{2}}}+c$, with WS and accelerations as dependent variable *y* and age as independent variable *x* (Figure 2).

As expected, WS increased quickly in the first years of life, based on the rise in anthropometric dimensions, and slowly decreasing for the remainder of life after the teenage years. In details, RMS_AP_ declined rapidly in the first years of life, then being progressively reduced, despite an increase toward the end of life. RMS_CC_ decreased in childhood, becoming progressive and smooth for the remainder of life. The coefficient of determinations (*R*^2^) of all four fits graphed in Figure 2 was between 0.34 and 0.53, showing goodness of fits from moderate to good (Cohen, 1988). The lower value was for the RMS_LL_. In this case, in fact, the bi-exponential trend lacks to fit the initial abrupt decrement occurred in childhood, well matching the data only after 7 years. It is noteworthy that lateral acceleration rose again in persons older than 70 toward values similar to those of children.

Because most studies have compared two groups of subjects, usually young versus elderly persons, little attention has been paid to the ages between 10–20 and 35–60 years. Nevertheless, we noted clear trends of rapid improvement in upright gait stability in the first period of life (reduction of RMS) despite the improvements in WS, and a loss of stability (increase of RMS) in older ages.

**Figure 3:**
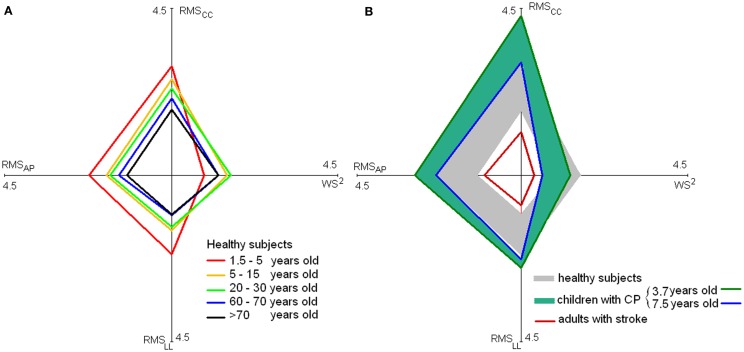
**Stability and velocity of walking**. Values of mean RMSs [along the antero-posterior (AP), latero-lateral, (LL) and cranio-caudal (CC) axes] and WS^2^ for healthy subjects at various ages [**(A)**, on the left] and for children with cerebral palsy and adults with stroke [**(B)**, on the right]. In the latter graph, the large gray rhomboidal area is formed by joining the extremes of values of healthy subjects [reported in **(A)**], and blue–green rhomboidal area by joining the extremes of values of children with cerebral palsy.

Because the quadratic relationship between WS and the three RMSs (Menz et al., 2003), a linear relationship exists between WS^2^ and RMSs as shown by Mazzà et al. (2008). Hence, more regular quadrilateral shapes are expected for healthy subjects than for toddlers or patients for the plots reported in Figure 3A and formed by the mean values of WS^2^ and of the three RMS values (obtained averaging the values over studies). On the left plot of this figure these quadrangles were reported for healthy subjects aged 1.5–5, 5–15, 20–30, 60–70, and over 70 years. Five of the studies in Table 1 were not taken into account in generating this figure [two due to the instruments that were used (Mazzà et al., 2008; Iosa et al., 2010), two due to the absence of data along all the three body axes (Lamoth et al., 2011; Senden et al., 2012), and one due to subjects with a mean age of 35 years (Henriksen et al., 2004)].

The quadrilateral shape of younger children trended toward higher acceleration and lower speed. Older children and young adults generated a more rhomboidal shape, with lower acceleration and increased speed. Finally, elderly showed a reduction both in terms of speed and accelerations.

The reduction of gait instability was fast both along AP- and LL-axes during gait development, followed by a slight sloped trend, positive along AP and negative along LL-axis. Finally, after 65 years of age, an abrupt increase of instability occurred. Unfortunately, only sporadic data are available for age ranges between 10 and 20 years and between 40 and 60 years included; thus, upright gait stability in these ranges requires further investigation. On the right side of Figure 3, on the quadrilateral shape of healthy subjects were superimposed those of children with cerebral palsy (a range was shown to take into account the different ages) and subjects with stroke extracting the data by the relevant studies reported in Table 2.

## Conclusion

Upright gait is a peculiar characteristic of human beings. This feature usually needs approximately 1 year to evolve but continues to improve during development. Body growth implies the possibility of increasing WS and the need to manage higher instabilities. In challenging environments or with existing physical impairments, the easiest solution to maintain upright gait stability is to reduce WS. This approach is also used by elderly subjects to compensate for the effects of aging on sensorimotor and cognitive functions.

The decline of upright gait stability with age is a slow but progressive process that increased drastically after 70 years. Pathologies can abruptly increase this loss proportionally to the severity of the impairment. The increase in upper body accelerations may be a direct effect of the impairment or a mixed result of impairment and compensatory strategies, as in children with atypical development (such as those with cerebral palsy) and persons with a progressively impairing pathology (such as muscular dystrophy).

A decrease in upright gait stability also exposes subjects to the risk of falls, which is particularly dangerous in the elderly. The development of low-cost and easy-to-use wearable sensors can facilitate the introduction of instrumented movement analysis in clinical settings (Iosa et al., 2012e). The clinical monitoring of upper body accelerations may provide a quantitative and hence objective outcome measure of the benefit of a rehabilitative pathway, evaluating the same parameters measured by vestibular system for maintaining balance.

Wireless and wearable technology have now provided us the possibility to quantify a feature, the importance of which was already noted in ancient time, when Aristotle wrote: “Man alone among all living beings walks erect, because his nature and his being are divine.”

## Author Contributions

Marco Iosa conceived this review, analyzed the data, and wrote the first draft. Augusto Fusco and Giovanni Morone provided a critical revision of manuscript for important clinical intellectual content. Stefano Paolucci supervised the entire work and obtained funding.

## Conflict of Interest Statement

The authors declare that the research was conducted in the absence of any commercial or financial relationships that could be construed as a potential conflict of interest.
